# Light chain plays a role in neutralizing antibody in b12 H chain knock-in mouse

**DOI:** 10.1186/1742-4690-9-S2-P98

**Published:** 2012-09-13

**Authors:** T Ota

**Affiliations:** 1 The Scripps Research Institute, La Jolla, CA, USA

## Background

HIV vaccine design has focused on epitopes seen by broadly neutralizing antibodies (bNAbs). The key to developing a robust vaccine capable of eliciting bNAbs is to identify the epitopes appropriate for incorporation into vaccines and to exclude irrelevant or harmful epitopes.

## Methods

To assess bNAb B cell responses, we have developed series of bNAb B cell lines and b12 ‘knock-in’ mouse.

## Results

Antibodies targeting the CD4 binding site, including PGV04 and b12, expressed higher levels of surface Ig compared to MPER targeting antibody 4E10. Correlating with the cell line data, in b12 heavy chain knock-in mice, B cells could develop robustly (this was not the case in 4E10 mice). 10-20% of b12 transgenic B cells bound to YU2 gp120 monomer and lacked expression of CD93, indicating that they matured normally and were non-anergic. Upon immunization with YU2 trimer, b12 mice exhibited strong gp120-specific IgG responses. Gp120-binding cells used Vk10-96 and Vk19-93, but were skewed to usage of Jk2 and Jk4, which are normally used less frequently than Jk1 and Jk5. Cells in the gp120- non binding fraction frequently expressed Vk1-135. Interestingly, the CDR1 length of Vk1-135 is 11 amino acids in length, whereas in Vk10-96 and Vk19-93 genes it is 6 amino acids, suggesting that a short CDR1 is important for gp120 binding. Several anti-gp120 hybridomas were established from LPS-stimulated B cells. However, compared to the original b12 antibody, these hybridoma cells did not show strong neutralization activity.

## Conclusion

The antibodies targeting CD4 binding site are apparently not autoreactive, supporting the notion that this is a desirable epitope for vaccine target. B12 H chain knock in mouse data showed light chain is important for bNAb function. B12 knock in mice and recently generated b12 germline strains are suitable for vaccine study.

